# Suitability of Chitosan Scaffolds with Carbon Nanotubes for Bone Defects Treated with Photobiomodulation

**DOI:** 10.3390/ijms23126503

**Published:** 2022-06-10

**Authors:** Samantha Ketelyn Silva, Ana Maria Guzzi Plepis, Virginia da Conceição Amaro Martins, Marilia Marta Horn, Daniela Vieira Buchaim, Rogerio Leone Buchaim, André Antônio Pelegrine, Vinícius Rodrigues Silva, Mateus Hissashi Matsumoto Kudo, José Francisco Rebello Fernandes, Fabricio Montenegro Nazari, Marcelo Rodrigues da Cunha

**Affiliations:** 1 Department of Morphology and Pathology, Jundiaí Medical School, Jundiaí 13202-550, Brazil; ra1904001@g.fmj.br (S.K.S.); vinnyeduca@gmail.com (V.R.S.); mateushmk@hotmail.com (M.H.M.K.); josefranciscorf@gmail.com (J.F.R.F.); professornazari@gmail.com (F.M.N.); 2 Interunits Graduate Program in Bioengineering (EESC/FMRP/IQSC), University of Sao Paulo (USP), Sao Carlos 13566-590, Brazil; amplepis@iqsc.usp.br; 3 Sao Carlos Institute of Chemistry, University of Sao Paulo (USP), Sao Carlos 13566-590, Brazil; virginia@iqsc.usp.br; 4 Physical Chemistry of Nanomaterials, Institute of Chemistry and Center for Interdisciplinary and Nanostructure Science and Technology (CINSaT), University of Kassel, 34109 Kassel, Germany; mariliahorn@gmail.com; 5 Postgraduate Program in Structural and Functional Interactions in Rehabilitation, Postgraduate Department, University of Marilia (UNIMAR), Marília 17525-902, Brazil; danibuchaim@alumni.usp.br; 6 Teaching and Research Coordination of the Medical School, University Center of Adamantina (UniFAI), Adamantina 17800-000, Brazil; 7 Department of Biological Sciences, Bauru School of Dentistry (FOB/USP), University of Sao Paulo, Bauru 17012-901, Brazil; rogerio@fob.usp.br; 8 Graduate Program in Anatomy of Domestic and Wild Animals, Faculty of Veterinary Medicine and Animal Science, University of Sao Paulo, Sao Paulo 05508-270, Brazil; 9 School of Dentistry, Faculdade Sao Leopoldo Mandic, Campinas 13045-755, Brazil; pelegrineandre@gmail.com

**Keywords:** bone regeneration, low-level laser therapy, chitosan, carbon nanotubes, immunohistochemistry, photobiomodulation

## Abstract

Biomaterials have been investigated as an alternative for the treatment of bone defects, such as chitosan/carbon nanotubes scaffolds, which allow cell proliferation. However, bone regeneration can be accelerated by electrotherapeutic resources that act on bone metabolism, such as low-level laser therapy (LLLT). Thus, this study evaluated the regeneration of bone lesions grafted with chitosan/carbon nanotubes scaffolds and associated with LLLT. For this, a defect (3 mm) was created in the femur of thirty rats, which were divided into 6 groups: Control (G1/Control), LLLT (G2/Laser), Chitosan/Carbon Nanotubes (G3/C+CNTs), Chitosan/Carbon Nanotubes with LLLT (G4/C+CNTs+L), Mineralized Chitosan/Carbon Nanotubes (G5/C+CNTsM) and Mineralized Chitosan/Carbon Nanotubes with LLLT (G6/C+CNTsM+L). After 5 weeks, the biocompatibility of the chitosan/carbon nanotubes scaffolds was observed, with the absence of inflammatory infiltrates and fibrotic tissue. Bone neoformation was denser, thicker and voluminous in G6/C+CNTsM+L. Histomorphometric analyses showed that the relative percentage and standard deviations (mean ± SD) of new bone formation in groups G1 to G6 were 59.93 ± 3.04a (G1/Control), 70.83 ± 1.21b (G2/Laser), 70.09 ± 4.31b (G3/C+CNTs), 81.6 ± 5.74c (G4/C+CNTs+L), 81.4 ± 4.57c (G5/C+CNTsM) and 91.3 ± 4.81d (G6/C+CNTsM+L), respectively, with G6 showing a significant difference in relation to the other groups (a ≠ b ≠ c ≠ d; *p* < 0.05). Immunohistochemistry also revealed good expression of osteocalcin (OC), osteopontin (OP) and vascular endothelial growth factor (VEGF). It was concluded that chitosan-based carbon nanotube materials combined with LLLT effectively stimulated the bone healing process.

## 1. Introduction

Bones are composed of dense and rigid connective tissue, but they are highly dynamic, and constantly remodeled [1]. Therefore, bone tissue is predisposed to different types of injuries caused by trauma, tumors, abnormalities and physiological imbalances, resulting in bone loss with difficulty in spontaneous regeneration [2,3]. Thus, considering these circumstances and the increasing demand for orthopedic and trauma treatment due to fractures caused by road accidents, it becomes necessary to develop or improve artificial bone substitutes, given the limitations of using autologous grafts and their postoperative complications, such as local infections and severe pain in the donor area [4,5]. In this scenario, it becomes necessary to study new biomaterials that mimic the components of bone tissue or have the ability to act as a scaffold that allows optimal adhesion and cell proliferation for the growth of new tissue [1,6].

Natural polymers have been highlighted because, when used to manufacture scaffolds, they can promote a support structure for tissue formation [7]. On the other hand, biopolymers that show similar composition as the extracellular matrix (ECM) can also be used to develop scaffolds, such as the polysaccharide chitosan. This polysaccharide can be obtained from squid pens of the species *Doryteuthis* spp., and it can be used in the preparation of scaffolds with minimal immune response [8].

Chitosan is a natural polymer obtained through the deacetylation of chitin [9] and presents important properties, such as biodegradability and biocompatibility, besides antibacterial action [10,11]. Chitosan based scaffolds lack in mechanical strength and structural stability in hydrated conditions, and thereby limit its application for bone tissue regeneration [12,13,14,15]. Therefore, modifications or addition of other products can help to improve the properties of chitosan [16,17], such as natural bioactive injectable composites for the purpose of inducing angiogenesis (Chitosan/Hydroxyapatite/Heparin) and improving bone regeneration, these hydrogels being modified by the use of glycerol, as an additive and a pre-sterile production strategy to increase its mechanical resistance [18].

Chitosan electrospinning and its composite formulations for creating fibers in combination with other natural polymers have potential for use in tissue engineering. There is evidence of favorable properties and biocompatibility of chitosan electrospun composite biomaterials for a variety of uses [19]. Carbon nanotubes can also be used to improve chitosan scaffolds, which may represent an attractive option due to their tensile strength, high flexibility, promising bioactivity and good electrical conductivity [20,21,22].

Considering the search for a mechanism that accelerates the cell migration process associated with the use of these biopolymers, it is also possible to develop mechanisms capable of promoting protein synthesis and cell growth. Therefore, the photobiomodulatory effects of low-level laser therapy (LLLT) have been used in the treatment of bone lesions [23,24,25,26,27], since infrared wavelengths can stimulate the proliferation of osteoblasts and collagen deposition [28].

Due to the absence of studies on this new biomaterial, especially associated with photobiomodulation, this study aimed to investigate the effects of an experimental proto-col for the treatment of bone lesions using the implantation of scaffolds constructed with chitosan and carbon nanotubes, in addition to the application of the LLLT protocol.

## 2. Results

There were no complications that needed to be reported, and there was no disease or sign that strongly motivated the removal of an animal.

### 2.1. Structure of Biomaterials

Once dissolved in diluted acid solutions, the amino group present in the chitosan structure is protonated, which allows electrostatic interaction among other compounds or polymers. This study observes an electrostatic formation between the protonated chitosan and the functionalized carboxylic groups in the nanotube walls. Recently, we found out that the 0.25 mg of functionalized single-wall carbon nanotubes (f-SWCN) is the threshold concentration for the nanofiller in the chitosan host scaffolds. In fact, an increase in SWCN concentration could result in the formation of nano-cracks and have a negative effect on the mechanical properties [29]. Nevertheless, one of the characteristics of implant materials is to provide enough mechanical response in the damaged area, which is possible by the addition of nanofillers, such as carbon nanotubes. For that reason, the formulation containing 0.25 mg of carbon nanotubes was selected for the present study.

**Figure 1:**
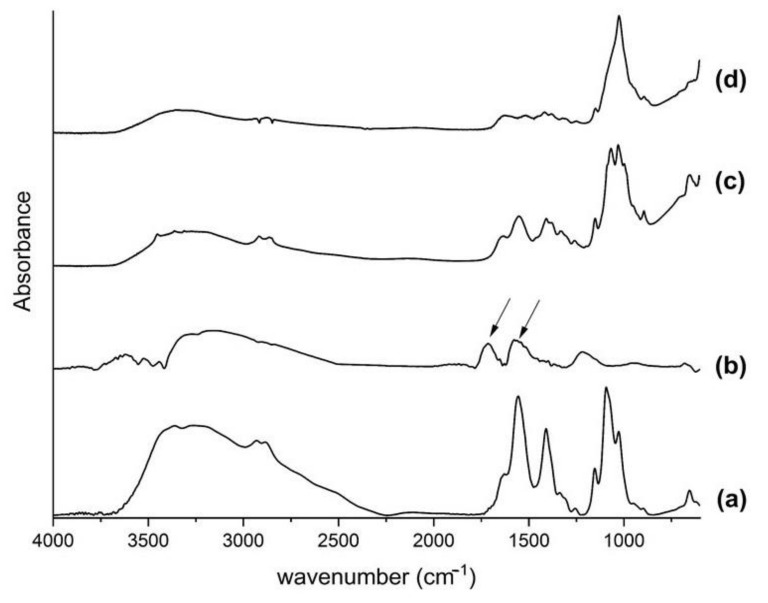
FTIR spectra of (**a**) chitosan, (**b**) carbon nanotubes, (**c**) CN25 and (**d**) CN25M.

The fingerprint region of the Fourier-transform infrared spectroscopy (FTIR) spectrum was used to analyse the characteristic bands of the chitosan and the functionalized carbon nanotubes. The molecular vibrations in this area are very accurate in characterizing chemical compounds. The chitosan spectrum (Figure 1a) showed the absorption bands characteristics of amides I and II at 1655 and 1560 cm^−1^, respectively. Additionally, -C-H banding at 1409 cm^−1^; C-O and C-O-C stretching vibrations at 1153, 1094 and 1026 cm^−1^, corresponding to the saccharide structure of chitosan. A typical band at 1716 cm^−1^ is attributed to the carboxyl groups, which evidence the effective functionalization of the carbon nanotubes, while the additional observed band at 1579 cm^−1^ is related to the aromatic C=C bond stretching of its structure, as indicated by the arrows (Figure 1b) [30].

The CN25 spectrum (Figure 1c) shows no changes or shifts in the characteristic bands, suggesting an electrostatic interaction between the components. The host polymer is comparatively in a higher concentration than the carbon nanotubes, overlapping its bands. The first evidence of the mineralization arises from the enhancement intensity of the PO_4_^3−^ band at 1024 cm^−1^. The sharp peak at this wavenumber is characteristic of phosphate compounds formed during the precipitation process (Figure 1d).

The nucleation and precipitation of calcium phosphate in scaffolds occur due to the ionic activity of calcium phosphate in solution and its stimulation to create favorable local conditions to allow the nucleation and growth of calcium phosphate. Indeed, calcium and phosphate ions diffuse into scaffolds and form nuclei of critical size for nucleation and further growth of calcium phosphate crystals [31]. Additionally, nucleated apatite crystals on three-dimensional scaffolds create a favorable environment for osteoconductivity.

**Figure 2:**
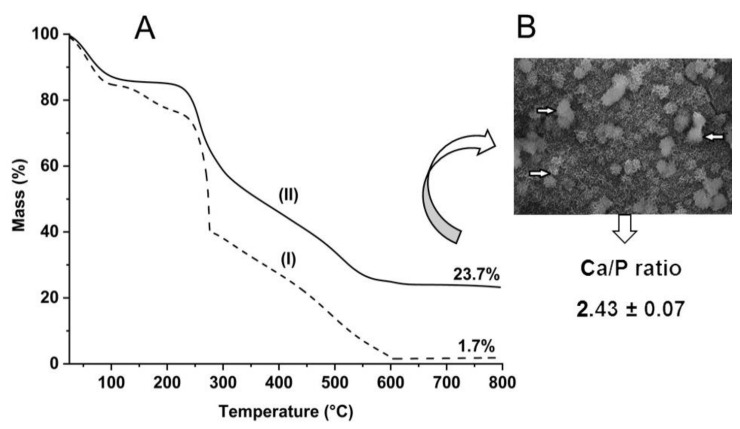
In (**A**), the thermogravimetric curves of: (I) CN25 and (II) CN25M. In (**B**), the SEM picture shows the calcium phosphate deposit used to determine the Ca/P ratio by energy dispersive X-ray analysis (EDX).

The quantification of deposited calcium phosphate was evaluated by thermogravimetric analysis, as the amount of residue at 750 °C refers to the inorganic material remaining after the decomposition of the organic phase (Figure 2A).

The curve for the non-mineralized scaffold (CN25) showed a residue of 1.7%, while the mineralized one (CN25M), exhibits a value of 23.7%. This result confirms the presence of an inorganic phase in the scaffold. It is interesting to notice that the processability of the materials has a considerable influence on the mineralization process. Compared to films, scaffolds are three-dimensional structures that allow nucleation and precipitation on the surface and in the internal network structure, increasing the amount of deposited calcium phosphate [32].

Using random spot areas (indicated by the arrows), as shown in Figure 2B, the determination of the Ca/P ratio was performed. The chemical analyses achieved by EDX stated the presence of calcium, phosphorous, and oxygen peaks. The calculated value was 2.43 ± 0.07, higher than expected for the theoretical hydroxyapatite value (1.67). This might be possible due to excess residual chloride from the mineralization process, and also, the deposited hydroxyapatite is calcium-rich amorphous, as described in the literature [33].

**Figure 3:**
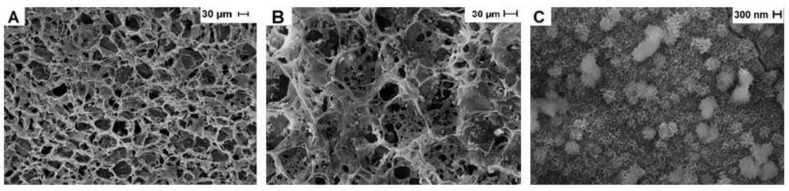
SEM images of (**A**), CN25; (**B**), CN25M at 500× magnification. In (**C**), CN25M at 25,000× magnification.

The SEM images using a 500× magnification show the presence of porous homogeneity distributed on the surface of the chitosan/carbon nanotube scaffold (Figure 3A). This property remains after the mineralization process (Figure 3B), and porous are still visible in the scaffold structure, which is a critical feature for the subsequent implant process. Additionally, at high magnification (25,000×, Figure 3C), aggregates of calcium phosphate crystals are observed in a spherical shape, a similar morphology of that class of inorganic deposits [34]. An effective mineralization process is evidenced by the formation of a bioactive compound, with the ability to induce new bone tissue formation. The pore size of the scaffolds was determined by the analyses of the SEM structures. The calculated value for CN25 was 20.6 ± 3.2, while for CN25M 13.5 ± 3.3. This result revealed the decrease in pore size after the mineralization process due to the calcium phosphate deposits along the surface of the collagen fibers.

The mineralized sample was previously examined by X-ray diffraction, showing the characteristic peaks of a poor crystalline state of hydroxyapatite and a crystallite diameter of 15 ± 1 nm [32].

The swelling characteristics of the scaffolds are directly related to their composition and affect the stability and the in vivo performance of the scaffolds, especially those related to the vascularization process.

**Figure 4:**
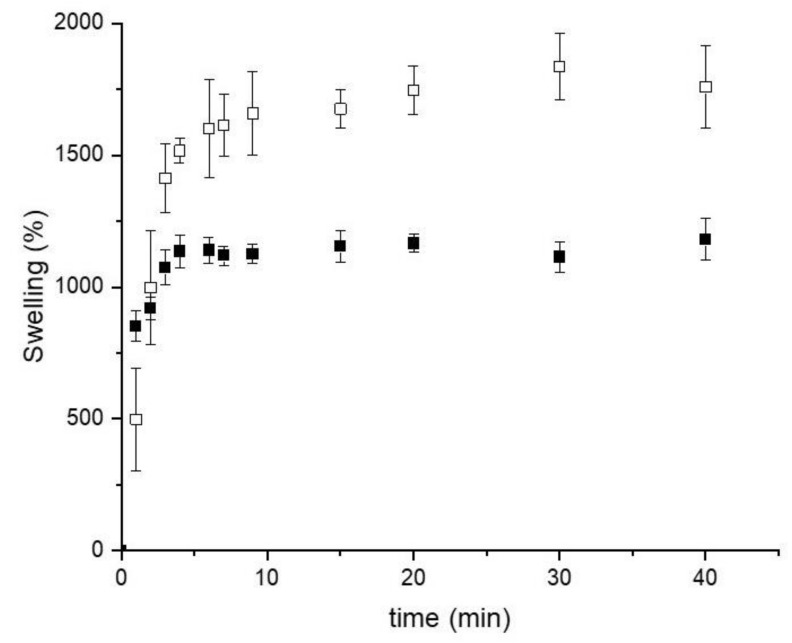
Swelling curves of (**☐**) CN25; (■) CN25M, performed in PBS pH 7.4.

Figure 4 shows the swelling behavior of CN25 and CN25M in PBS pH 7.4. In both cases, the absorption of the buffer is fast, leading to percentage values higher than 1000%. This characteristic is typical for three-dimensional scaffolds composed of polymeric networks capable of swelling without the rupture of their structure. The measurement was carried out for 40 min, during which the buffer absorption equilibrium achievement was observed. A maximum swelling percentage of 1716% (CN25) and 1179% (CN25M) was noticed. Even though both samples exhibited a high swelling percentage, the mineralization process reduced the amount of PBS absorbed by the scaffold, probably by the reduction in the pore size. The reduction in the swelling property is expected in mineralized chitosan scaffolds, as observed in previous studies described in the literature [35].

### 2.2. Macroscopic and Radiological Analysis of the Bone Lesion

**Figure 5:**
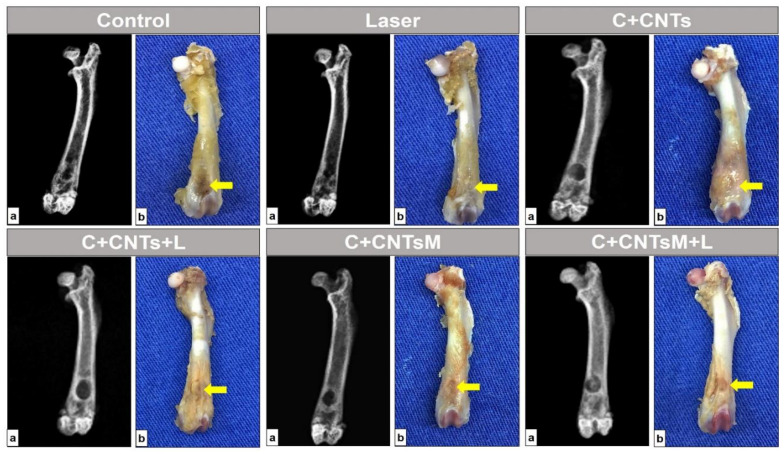
Radiological (**a**) and macroscopic (**b**) images of the left femur of rats in the study groups. Bone defects are indicated by the yellow arrows. Bone integrity is observed without injuries or secondary complications, as well as the presence of a bone defect that has not completely regenerated.

In all surgical areas of the animals evaluated in this study, no signs of anatomopathological changes were observed, such as local inflammatory process, bone non-union, ulcerative lesions, purulent secretions, cysts, infection suggestive of osteomelitis characterized by radiographic bone rarefaction or abnormal mass growth tissue with neoplastic characteristics. The anatomical architecture of the femur was maintained in all animals evaluated, with no secondary fractures or pseudarthrosis resulting from post-surgical complications. The normality pattern of the radiological characteristics of the femur, such as the radiopaque cortical margins of the bone and the radiotransparency of the medullary canal, could also be observed. However, the bone defects were not completely closed in the studied groups (Figure 5).

### 2.3. Morphology of the Bone Lesion Area

In all the groups studied, new bone formation was noted, projecting from the original bone from the edges of the lesion and towards the center of the surgical area. However, the morphology of this bone presented different characteristics for each group of animals.

**Figure 6:**
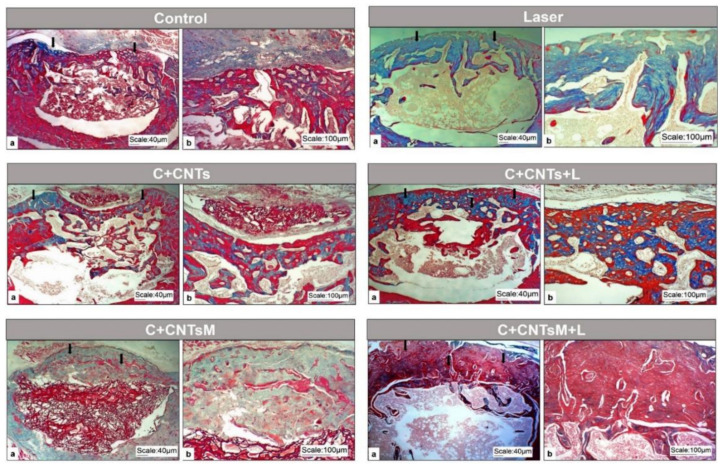
Optical microscope images of the bone defect created in the distal metaphysis of the femur of rats in the study groups, (**a**) panoramic view and (**b**) detailed view. Defect areas are indicated by black arrows. Note the neoformed bone in the surgical area, is more dense and voluminous in G6/C+CNTsM+L. Masson trichrome.

In the control group (G1/Control), the young bone acquired a thinner and irregular aspect, with histological characteristics of immature tissue, with several cavities inside. In the animals treated only with LLLT (G2/Laser), subperiosteal bone formations extended along the lesion area, with some long trabeculae arranged in different directions. The group that received chitosan/carbon nanotubes (G3/C+CNTs) had a more porous bone formation, with bone cavities inside and remnants of the scaffolds surrounded by connective tissue. In the chitosan/carbon nanotubes group that received the LLLT (G4/C+CNTs+L), the bone neoformation filled the entire extension of the bone lesion, thus uniting the edges of the lesion extremities. When mineralization was added, it was observed that the G5/C+CNTsM group presented remnants of the biomaterial centralized close to the medullary canal, in addition to a neoformed bone tissue of a dense nature and with few cavities in its interior. In the last group evaluated, which received the scaffolds and the LLLT (G6/C+CNTsM+L), bone formation was relatively higher than in the other groups, with a thicker and compact aspect able to fill the entire lesion area (Figure 6).

**Figure 7:**
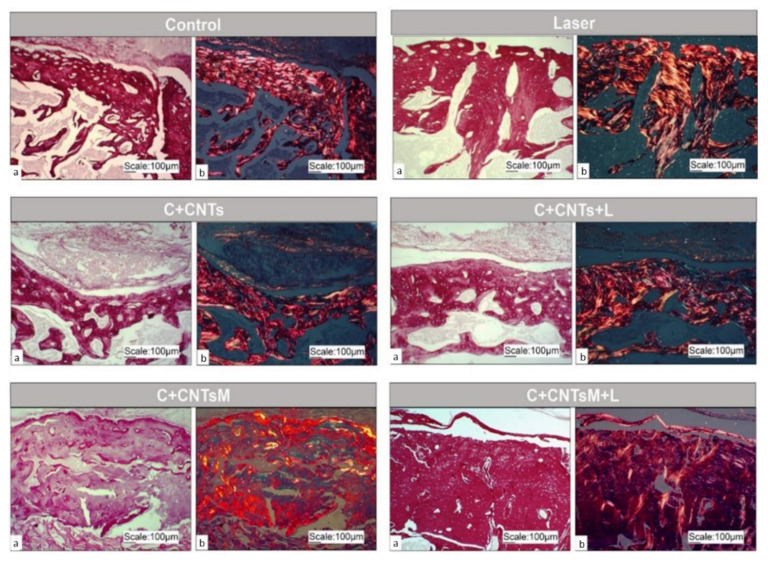
Picrosirius red optical microscope images in optical light (**a**) and respective polarized light (**b**) of the study groups, 10× magnification. Birefringence of the extracellular matrix is observed in the area of the bone lesion.

Through picrosirius red staining and polarized light, the birefringence of collagen in the extracellular matrix of the tissue present in the surgical area was observed in all groups evaluated (Figure 7).

### 2.4. Histomorphometric and Statistical Analysis of the Bone Volume Formed in the Surgical Area

**Figure 8:**
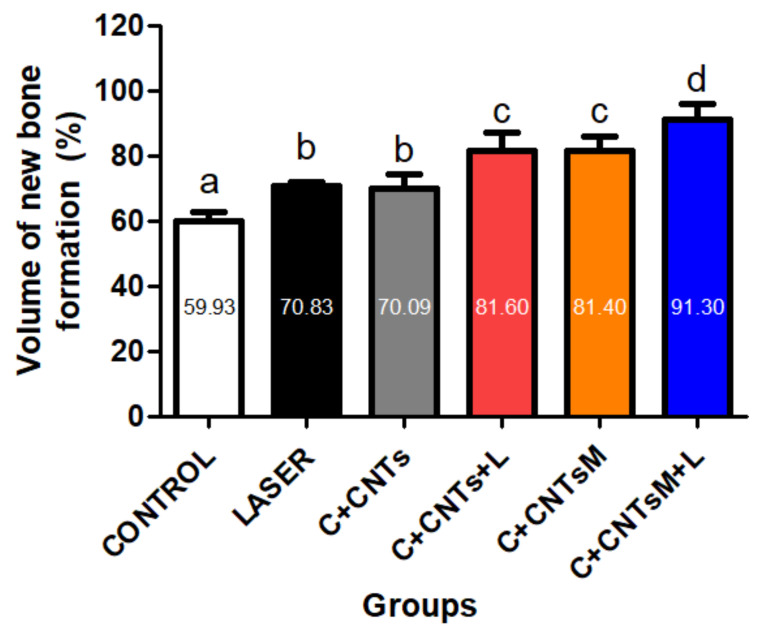
Bone volume (%) formed in the defect area. The means and standard deviation of the relative percentage volume of newly formed bone in the femoral defects in the study groups were: 59.93 ± 3.04 (G1/Control), 70.83 ± 1.21 (G2/Laser), 70.09 ± 4.31 (G3/C+CNTs), 81.6 ± 5.74 (G4/C+CNTs+L), 81.4 ± 4.57 (G5/C+CNTsM) and 91.3 ± 4.81 (G6/C+CNTsM+L), respectively. Different lowercase letters indicate a significant difference between the groups. (a ≠ b ≠ c ≠ d; *p* < 0.05).

The means and standard deviation of the relative percentage volume of newly formed bone in the femoral defects in the study groups were, respectively: 59.93 ± 3.04a (G1/Control), 70.83 ± 1.21b (G2/Laser), 70.09 ± 4.31b (G3/C+CNTs), 81.6 ± 5.74c (G4/C+CNTs+L), 81.4 ± 4.57c (G5/C+CNTsM) and 91.3 ± 4.81d (G6/C+CNTsM+L) (a ≠ b ≠ c ≠ d; *p* < 0.05). In the comparative statistical analyses between the groups, it was observed that the treated experimental groups (G2/Laser to G6/C+CNTsM+L) showed satisfactory results, as they presented higher values when compared to the control group (G1/Control). Regarding the LLLT protocol, it was noted that the parameters used were essential for bone formation since G6/C+CNTsM+L showed greater bone volume compared to G5/C+CNTsM, as was also observed in the comparison between G4/C+CNTsM+L and G3/C+CNTs. In addition, it was found that there was a significant difference in formed bone volume in the animals that received mineralized scaffolds compared to the non-mineralized scaffold groups, as observed in the comparison between G3/C+CNTs vs. G5/C+CNTsM (Figure 8).

### 2.5. Immunohistochemical Analysis

**Figure 9:**
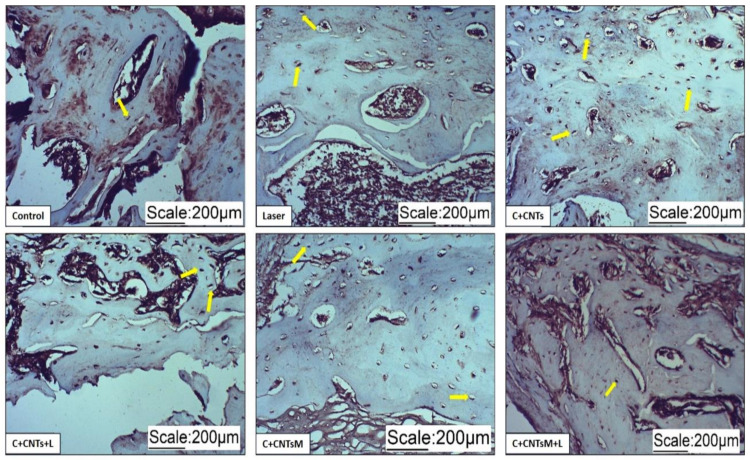
Optical microscope images of osteocalcin immunostaining of the groups studied. Osteocytes were identified by the expression of osteocalcin. The yellow arrows indicate the labeling of osteocytes.

**Figure 10:**
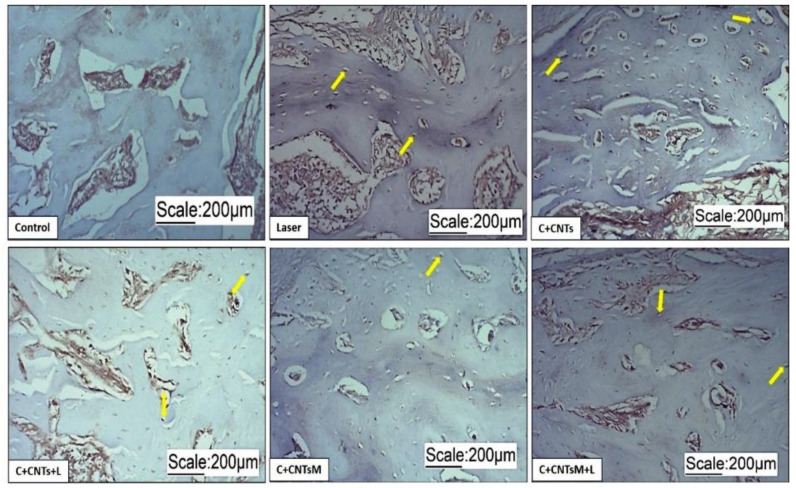
Optical microscope images of osteopontin immunostaining of the groups studied. Osteocytes were identified by the expression of osteopontin (yellow arrows).

**Figure 11:**
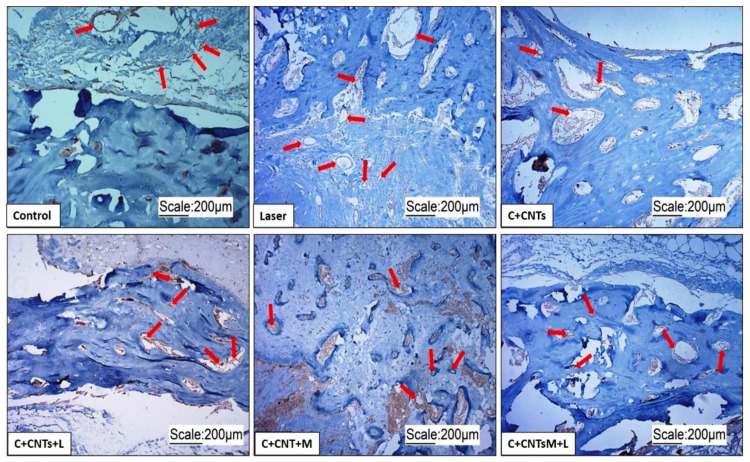
Optical microscope images of VEGF immunostaining of the groups studied. Note the bone defect and the presence of blood vessels (red arrows) distributed along the cortical bone.

In the newly formed bone matrix of the surgical area, it was possible to observe the formation of osteocytes due to the expression of osteocalcin (OC) and osteopontin (OPN). In the groups treated with LLLT, a more organized arrangement of osteocytes was observed (Figure 9 and Figure 10). Regarding the expression of vascular endothelial growth factor (VEGF), the formation of blood vessels was also noted in the surgical areas (Figure 11).

## 3. Discussion

The treatment of bone injuries is still a challenge [36] for regenerative medicine, and, thus, there is a need to manufacture biomaterials and improve treatment protocols that enable bone replacement quickly and safely [37], overcoming the limitations of conventional techniques of autograft and allograft, which can lead to unwanted infections and donor site morbidity [38]. However, some factors must be considered in the formulation of these products, such as their biocompatibility, surface type and porosity, mechanical strength, physical-chemical and biological composition, and the three-dimensional arrangement that allows osteoconduction [39]. The bone resorption capacity is also important as the degradation of the material is expected to occur as new bone tissue grows [10,40]. Thus, polymers stand out among the various materials that present these characteristics due to their greater bioactivity and the non-release of cytotoxic products during degradation [41,42].

Chitosan is a favorable biopolymer for tissue engineering, mainly due to its biodegradable, non-toxic, bioactive, biocompatible, non-immunogenic, and antibacterial features. Additionally, it shows a similar structure to glycosaminoglycans (GAGs) and proteoglycans found in the extracellular matrix (ECM), which are associated with essential physiological functions in the tissue [43,44]. Due to its cationic nature, chitosan interacts electrostatically with anionic molecules. This approach is usually applied to outcome some limitations of the chitosan, e.g. the mechanical properties. Consequently, the combination of chitosan and carbon nanotubes is an alternative to enhance the mechanical and structural properties of the material. Scaffolds should be capable of supporting tissue formation and also cooperate in the tissue repair permanently or temporarily. For that reason, the scaffold used in this study may be an alternative material to restore bone defects [45,46]. Scaffolds for bone tissue regeneration should have crucial characteristics, such as the interconnected pore structure to allow vascularization and the transport of nutrients, and adequate mechanical properties. Additionally, the mineralization process provides the deposit of calcium phosphate inorganic phase, which is essential in the biomineralization process during the repair of the damaged tissue [47].

Carbon nanotubes are considered a promising material due to their mechanical and magnetic properties [48]. Türk et al. (2018) evaluated the performance of carbon nanotube composites with chitosan/hydroxyapatite/collagen, and the results were satisfactory for bone tissue [49]. Cunha et al. (2017) showed the biocompatibility of chitosan/carbon nanotube scaffolds implanted in the calvaria of rats, as no macroscopic and radiological changes from inflammatory processes were observed in the surgical area [50]. In agreement with these data, an investigation performed on the femur of rats also did not find signs of the formation of a granulomatous foreign body that would indicate immunological rejection or any inflammatory processes after the grafting of biomaterials [51]. These results are attributed not only to the antimicrobial activity of chitosan but also to the physicochemical process of the manufacturing of these biopolymers [51]. These biocompatibility characteristics with the host tissue demonstrates the applicability of these biopolymers, however, a scaffold must also promote sufficient osteogenesis to initiate a bone repair process.

Bone formation observed in the group that received chitosan/carbon nanotubes (G3/C+CNTs) was statistically higher when compared to the control group (G1/Control), with the advantage to show a denser and more regular appearance. Thus, it is evident that these effects on bone tissue are due to chitosan properties, such as biodegradation [46] and the ability to form porous structures, additionally to the feature of the surface capability of promoting cell growth [8,52]. Accordingly, the histomorphometric data of this research points to chitosan as a promising option for use in the manufacture of scaffolds, but the isolated use of chitosan can compromise some essential functions of scaffolds, such as mechanical strength [53]. For this, the addition of composites that can improve biomaterials becomes interesting, and, in this way, carbon nanotubes can be a viable alternative since they are able to modulate the behavior of cells [54].

In a study that evaluated the effect on bone repair in rat tibiae grafted with carbon nanotubes and sodium hyaluronate, it was noted that the grafted groups showed greater osteoregenerative potential than the control group [55]. Xu et al. (2019) showed that multi-walled carbon nanotubes composite scaffolds prepared by freeze-drying promoted cell proliferation in vitro [56]. Thus, the addition of nanotubes to chitosan can potentiate cellular interactions [57], thus supporting the hypothesis that chitosan associated with carbon nanotubes can stimulate the bone repair process, as demonstrated in the histomor-phometric analyses of this research [58,59,60,61,62,63,64].

The mineralization of scaffolds can further potentiate the osteogenesis process [65], however, contradictory results were demonstrated by Munhoz et al. (2018), who reported that the mineralization of collagen and chitosan sponges did not stimulate bone neoformation sufficiently for tissue repair [66]. However, it must be considered that the experimental procedure was performed on the calvaria of rats, which is not subject to biomechanical load by muscle action. Therefore, other variables must also be considered in studies with scaffolds, such as the mineralization concentration of the materials, as well as the type of bone to be studied. Therefore, in the morphometric analysis of the surgical areas, it was observed that the groups that received the mineralized scaffolds presented a statistically higher bone volume, in relation to the groups that received the non-mineralized scaffolds, as noted in the comparison between G5/C+CNTsM with G3/C+CNTs, as well as in the comparison between G6/C+CNTsM+L and G4/C+CNTs+L, in which LLLT treatment constitutes an additional variable. There is evidence in the literature demonstrating the positive action of polymers [47] and LLLT [67,68] in bone repair; however, there is no evidence of the combination of these two resources on osteogenic potential.

Thus, the analysis of the action of LLLT in the surgical area allowed us to verify the effectiveness of the photobiomodulation on the process of stimulation of bone repair, because the groups that received LLLT showed greater bone volume than their respective control groups, as observed in the comparisons between G2/Laser vs. G1/Control, G4/C+CNTs+L vs. G3/C+CNTs and G6/C+CNTsM+L vs. G5/C+CNTsM, and these promising data related to bone formation are due to the photomodulatory effects of LLLT.

Photobiomodulation consists of the use of light in non-ionizing forms, including lasers and LEDs of the visible spectrum and infrared for therapeutic purposes [69]. Its mechanism occurs from specific photoreceptors that are responsible for absorbing the light beam and for stimulating calcium transport and the mitochondrial respiratory chain, which results in increased synthesis of RNA, DNA, and cell cycle regulatory proteins essential for the significant cell proliferation and, consequently, for the production of ATP [70]. Furthermore, the light at a low radiation dose is absorbed by intracellular chromophores [71], initiating cell signaling, which may improve the differentiation of mesenchymal stromal cells into osteoblasts [72].

There is no consensus in the scientific literature regarding the laser parameters that would be effective for the treatment of bone lesions. However, in this research, the photobiomodulation protocol was created based on studies that obtained favorable results using the laser in the treatment of bone lesions. The 904 nm aluminum gallium arsenide pulsed diode laser is the most indicated in the therapeutic process of serious injuries due to its greater ability to penetrate tissues [73,74,75]. The literature also cites that this wavelength is capable of promoting an increase in blood flow in live rats, creating an ideal scenario for healing due to good tissue vascularization and efficient collagen deposition [76].

The literature describes an increase in growth factors in the laser-modulated bone formation process [26]. LLLT shows the ability to stimulate alkaline phosphatase activity and osteocalcin gene expression [77,78,79]. In this way, bone formation markers, such as OC and OPN, allow the assessment of the rate of bone formation and resorption, which are important during bone remodeling [80]. Osteopontin is a non-collagenous phosphoprotein involved in the biomineralization of bone tissue, being described as a structural element of the bone matrix [81]. Osteopontin is a secreted protein related to many events of bone metabolism and, therefore, it is used as a parameter to assess angiogenesis and cellular activity [82]. Osteocalcin participates as an important marker of bone formation, being found mainly during the final stage of differentiation of osteoblasts and in the initial stage of mineralization [83]. In the immunohistochemical analyses of this research, it was possible to observe the expression of these markers in the osteocytes of the neoformed bone in the surgical areas, as well as the presence of blood vessels through VEGF.

In this way, these morphological and immunohistochemical analyses show that the scaffold together with the LLLT protocol used in this research allowed for a more homogeneous organization of the extracellular matrix, and favored the maturation of the newly formed bone tissue, which was essential for tissue repair.

## 4. Materials and Methods

### 4.1. Experimental—Raw Materials

All solvents and salts were analytical grade and were used as received. Functionalized multi-walled carbon nanotubes (carboxylic acid > 8%) (f-MWCN) were purchased from Sigma-Aldrich and had the following characteristics: average diameter L of 9.5 nm × 1.5 μm.

Chitosan was extracted from squid pens (*Doryteuthis* spp.), as previously described [8]. Briefly, demineralization and deproteinization steps are necessary to isolate β-chitin and were carried out using dilute solutions of HCl and NaOH, respectively. Deacetylation of N-acetyl groups was performed using a concentrated sodium hydroxide solution (40% NaOH, *w*/*w*). The chitosan powder was obtained after the washing and drying processes. The degree of acetylation (9.05% ± 0.35) and molecular weight (4.4 × 10^5^ g mol^−1^) were determined by conductometric titration and capillary viscosimetry, respectively [8].

### 4.2. Sample Preparation

The chitosan powder was dissolved in 1% (*v*/*w*) aqueous acetic acid solution by magnetic stirring. A 1% (*w*/*w*) chitosan solution was used for the sample preparation. Then, 0.25 mg of functionalized MWCN was slowly added to the biopolymeric solution under mechanical stirring and then sonicated in an ultrasound bath (Único^®^ USC 1400 A) for 60 min to obtain an adequate homogeneous dispersion of the carbon nanotubes. Finally, the sample labeled CN25 was freeze-dried [34]. The alternate immersion method consists of alternating immersion cycles in calcium chloride and disodium phosphate solutions. As previously described [8], 0.12 mol·L^−1^ CaCl_2_ buffered with 0.05 mol·L^−1^ Tris buffer (pH 7.4) and a 0.06 mol·L^−1^ Na_2_HPO_4_ solution buffered with 0.05 mol·L^−1^ Tris buffer (pH 9.0) were the calcium and phosphate sources for the nucleation and precipitation of the apatite salt. Scaffolds were alternately placed in the solutions for 60 min and rinsed with deionized water during every change of the solution. All experiments were conducted at 37 °C. The freeze-dried mineralized scaffold, named CNM25, was prepared using two mineralization cycles.

### 4.3. Sample Characterization

FTIR analysis was performed for the powder chitosan and carbon nanotube. Spectra were obtained using Shimadzu IR Affinity^−1^ equipment at the interval of 2000–400 cm^−1^ and a resolution of 4 cm^−1^ and 64 scans. The CN25 and CN25M scaffolds were analysed in an FTIR-ATR (attenuated total reflectance) using a Bruker Tensor 27 FT-IR spectrophotometer, in the range of 4000–600 cm^−1^, resolution of 2 cm^−1^ and 64 scans.

Prior to the PBS swelling test, the scaffolds were placed in a desiccator in the presence of NaOH (s) for 24 h. The dried scaffolds were weighed (dry weight) and placed in 10 mL of the buffer solution. At predetermined times, they were removed from the liquid and weighed (wet weight), until achieving equilibrium (40 min). The process was carried out 5 times for each sample. The percentage of absorbed buffer (% absorption) was calculated by the equation: % absorption = [(wet weight − dry weight)/dry weight] × 100.

Thermogravimetric analysis was carried out in a TGA Q50 (TA Instruments^®^) from 25 to 800 °C at a heating rate of 10 °C min^−1^. A synthetic airflow of 90 mL·min^−1^ and sample weight of around 9 mg was employed for the measurement. The surface and calcium phosphate deposits were analysed by Scanning Electron Microscopy (SEM) (Zeiss LEO 440^®^, Cambridge, UK) with an Oxford detector (model 7060) LEO 440 at an accelerating voltage of 20 keV. For that, the scaffolds obtained were placed in stubs and coated with a thin layer of gold (6 nm) to improve the conductivity of the samples. Simultaneously, energy dispersive X-ray analysis (EDX) was used to determine the Ca/P ratio of the mineralized scaffold. For this analysis, the EDX equipment LEO 440^®^ (LEO Electron Microscopy Ltd., Cambridge, UK) Oxford detector mod. 7060^®^ (Oxford Instruments Inc., Concord, CA, USA) and 133 eV resolution were used. The selected standards were CaCO_3_, quartz, GaP, and Wollas (CaSiO_3_). Scaffold images of the surface (at a 500× magnification) were adopted to measure the pore size, where at least 40 pores of each sample were used. The ImageJ software was employed to calculate the average pore size.

### 4.4. Experimental Design

This research used 30 male Wistar rats (*Rattus norvegicus*) with 16 weeks of age and an average body weight of 400 g. This study was conducted according to the guidelines of the Declaration of Helsinki and approved by the Institutional Ethics Committee on Animal Experimentation of the Faculty of Medicine of Jundiaí, Sao Paulo, Brazil (CEUA/FMJ), and protocol code CEUA/FMJ No. 282/2016.

Furthermore, this experimental study was carried out according to the ARRIVE guidelines (Animal Research: Reporting of In Vivo Experiments) and based on the principles of the National Centre for the Replacement, Refinement, and Reduction of Animals in Research (NC3Rs) [84,85,86]. During the experimentation, the animals were monitored regarding the expression of pain by observing whether the animal was apathetic, depressed, aggressive, or overexcited, and these characteristics constitute variables in their usual behavior. Changes in walking, posture, appearance, or facial expression were also observed. Water and food consumption and clinical symptoms were also investigated.

**Figure 12:**
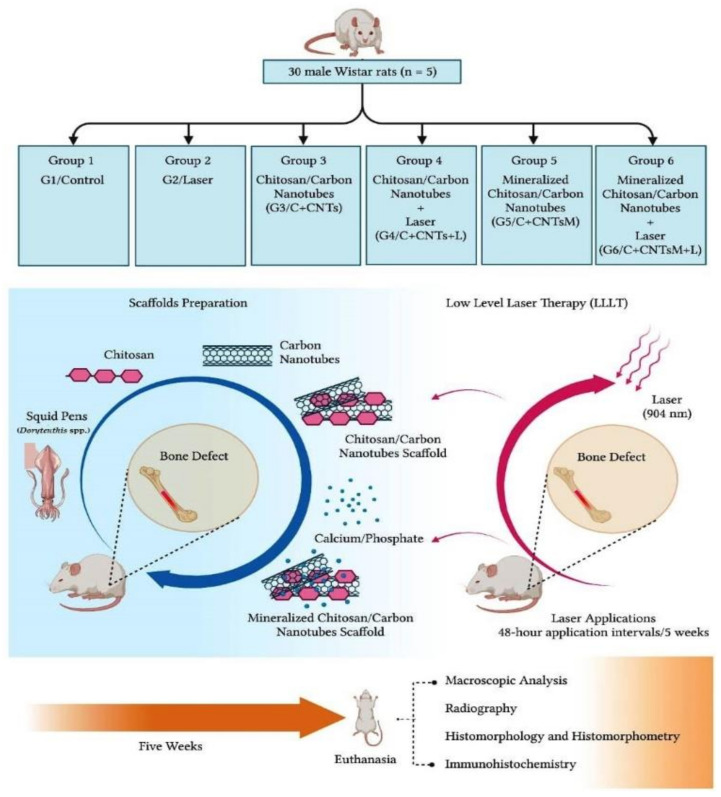
Design of the study groups and preparation of the scaffolds. Thirty male Wistar rats were divided into 6 groups: Control (G1/Control), Laser (G2/Laser), Chitosan and Carbon Nanotubes (G3/C+CNTs), Chitosan/Carbon Nanotubes/Laser (G4/C+CNTs+L), Mineralized Chitosan/Carbon Nanotubes (G5/C+CNTsM), Mineralized Chitosan/Carbon Nanotubes/Laser (G6/C+CNTsM+L). Analyses were performed after 5 weeks.

The animals were kept in the bioterium of the Faculty of Medicine of Jundiaí and submitted to create an experimental defect in the distal metaphysis of the femur. The rats were randomly distributed into 6 groups, without predetermined inclusion or exclusion criteria, according to the treatment received: Control (G1/Control), Laser (G2/Laser), Chitosan/Carbon Nanotubes (G3/C+CNTs), Chitosan/Carbon Nanotubes/Laser (G4/C+CNTs+L), Mineralized Chitosan/Carbon Nanotubes (G5/C+CNTsM), Mineralized Chitosan/Carbon Nanotubes/Laser (G6/C+CNTsM+L) (Figure 12).

The PBM protocol used for bone repair in this experimental protocol was based on previous experiments [23,24,26], which used a low-level laser in pulsed mode (Gallium Arsenide-GaAs), with a wavelength of 904 nm. The animals in groups G2/Laser, G4/C+CNTs+L and G6/C+CNTsM+L received this treatment of LLLT in the surgical area of the femur for 5 weeks, with application intervals of 48 h [87,88,89]. The complete photobiomodulation protocol is shown in Table 1.

### 4.5. Surgical Procedure

The animals were anesthetized with a solution of Xylazine (Vetaset^®^—Fort Dodge Saúde Animal Ltda., Campinas-SP, Brazil) and Ketamine (Dopalen^®^—Agibrands of Brazil LTDA, Campinas, Brazil) in the proportion of 1:1. This solution was applied at a dose of 1 mL/Kg of body mass, via the gluteal intramuscular route. After antisepsis with Riohex^®^ 4% degerming agent and trichotomy of the left hind law, a skin incision was made on its anterior face, reflecting the adjacent muscles to expose the distal metaphysis of the femur. Using a surgical drill of 3 mm in diameter, coupled to the pen of a mini motor (Eltec LB-100^®^, Eltec Elektronik AG, Mainz, Germany), a bone defect was made until reaching the medullary canal. In groups C+CNTs, C+CNTs+L, C+CNTsM, and C+CNTsM+L, bone defects were grafted with chitosan scaffolds/carbon nanotubes. A sample size of 2.5 mm was used for the in vivo test.

### 4.6. Macroscopic and Radiological Analysis of the Surgical Area

Five weeks after surgery, the animals were submitted to painless death induced by a high dose of intraperitoneal anesthetic, followed by a surgical procedure of pneumothorax. After death was confirmed by the absence of vital signs in each animal, disarticulation was performed in the hip and knee region, preserving the left femur. The surgical area was photodocumented, and the macroscopic conditions of anatomopathological reactions were evaluated. Then, the surgical areas of the animals were radiographed with an Odel 300 mA device, focus with 100 mA, time of 0.06 s, and radiation of 40 kV (X-ray tube voltage). Radiographic images were digitized using the Agfa system. The radiographic images were studied in order to assess the integrity of the bone defect in the surgical area.

### 4.7. Histological Analysis of the Surgical Area

The samples from the operated femurs were fixed in formaldehyde solution, then decalcified and subsequently submitted to routine histological techniques. From each sample, 5 μm-thick semi-serial microscopic sections were obtained. Histological slides were stained with Masson’s Trichrome for the evaluation and differentiation of the original bone and new bone formation. Picrosirius Red (saturated aqueous solution of picric acid added to 0.1 g of Syrian Red F3b, Sirius Red F3B-Bayer^®^) was also used to label the fibrillar constituents of the extracellular matrix in the area of the bone lesion through polarized light microscopy. The qualitative analysis of the histological sections of the surgical area allowed the evaluation of the bone failure repair process through the observation of the characteristics of the newly formed bone. A Nikon Eclipse E200 optical microscope was used, and the digital images were analysed through objective lenses with 4× and 10× magnification.

### 4.8. Histomorphometric and Statistical Analyses of the Bone Volume Formed in the Surgical Area

Slides stained with Masson’s Trichrome and a Nikon Eclipse E200^®^ light microscope with a 4× magnification objective were used in this analysis, and the slides were photographed. Using the Motic Image Plus 2.0 ML software, the volume of the bone defect area was delimited in the histological images, as well as the volume of the newly formed bone for each animal studied. From these values, the percentage of bone formed in the surgical area was obtained. These data were transcribed into the BioEstat 5.3 software, applying the ANOVA test followed by the Tukey’s test for statistical evaluation between the groups with a significance level of *p* < 0.05.

### 4.9. Immunohistochemical Analysis of Osteocalcin and Osteopontin Labeling

Anti-osteocalcin (Osteocalcin-BIOSS^®^ Rabbit Policlonal bs-4917R AGO9294227) and anti-osteopontin (Osteopontin Polyclonal Antibody^®^-bs-0026R) antibodies were used for this analysis. Initially, the slides were deparaffinized in xylol and hydrated. Endogenous peroxidase was blocked with hydrogen peroxide for 10 min, followed by washing in running distilled water. The primary antibody (previously standardized) was diluted in bovine serum albumin (BSA). The slides were placed with the antibody on the sections in a humid chamber and incubated overnight in the refrigerator. Subsequently, they were washed with phosphate-buffered saline (PBS) pH 7.4 and incubated in a humid chamber with a secondary antibody (30 min in an incubator at 37 °C). Thereafter, the slides were washed with PBS, stained with diaminobenzidine (DAB) chromogen (5 min), and counterstained with Hematoxylin for approximately 1 min. In order to remove excess dye, the slides were washed in running water, and the sections were dehydrated and placed in the incubator to dry completely. Finally, the sections were covered with coverslips using Entellan (Merck) [25].

### 4.10. Immunohistochemical Analysis—VEGF

Immunohistochemistry was performed as previously described [90], on 3 µm whole sections, using VEFG (BioSB^®^—BSB 6053—Clone RBT-VEGF). Briefly, sections were deparaffinized with xylene and dehydrated in alcohol. Endogenous peroxidase was blocked with 3% hydrogen peroxide. Antigen retrieval was achieved by immersing slides in citrate buffer, pH 5.8 per 30 min, in a commercially available pressure cooker (Pascal^®^, Dako, Carpenteria, CA, USA). The sections were incubated in a humid chamber with the specific primary antibodies (1:200) at 37 °C, for 40 min, and after, at 4 °C overnight. Then, slides were washed in PBS 0.1 mol L^−1^, pH 7.4 and a secondary antibody was used (kit Novolink Leica^®^—Cod. RE 7280-K) during 30 min, at 37 °C. The Advance HRP Detection System^®^ (Dako K3467) was used according to the manufacturer protocol. Finally, the slides were counterstained with Harris’ hematoxylin for 30 s, dehydrated, and mounted in Entellan^®^ (Merck, Darmstadt, Germany). Internal and external, positive and negative controls were used in order to validate the reactions [87].

## 5. Conclusions

Based on histomorphometric and immunohistochemical evidence of bone formation in the surgical area, it was concluded that the chitosan/carbon nanotube scaffolds contribute to the regeneration of lesions in long bones, such as the femur of rats, and that additional factors, such as mineralization and LLLT, can stimulate this bone repair process. Thus, this scaffold can be considered as an alternative in trauma therapies that require surgical intervention, with bone grafting and rapid rehabilitation processes, as well as in the advancement of new studies of tissue engineering in polymeric biomaterials and photobiomodulators for bone regeneration.

## Figures and Tables

**Table 1:** Photobiomodulation protocol.

Parameter	Unit/Description
Type of laser	GaAs (gallium-arsenide) Endophoton LLT 1307 (KLD^®^ Biosistemas Equip. Elet. Ltda, Amparo, Brazil)
Output power	70 mW
Wavelength	904 nm (APLP 904, KLD^®^ Biosistemas Equip. Elet. Ltda, Amparo, Brazil)
Pulse width	100 ns
Power density	7000 mW/cm^2^
Energy density	616 J/cm^2^
Energy per point	6.16 J
Beam area	0.01 cm^2^
Total energy applied	18.48 J
Beam type	Punctual
Emission mode	Continuous
Form of application	Three points in the surgical area
Irradiation duration	88 s per point
Total time of each application	264 s
Treatment time	Immediately after the surgery and three times a week for five weeks

GaAs = gallium-arsenide; mW = milliwatts; nm = nanometer; ns = nanosecond; mW = milliwatts/centimeter^2^; J/cm^2^ = joules/centimeter^2^; cm² = centimeter^2^; J = joules.

## Data Availability

The data presented in this study are available on request from the corresponding author.
